# Genome-Wide Analysis of the *COBRA-Like* Gene Family Supports Gene Expansion through Whole-Genome Duplication in Soybean (*Glycine max*)

**DOI:** 10.3390/plants10010167

**Published:** 2021-01-16

**Authors:** Sara Sangi, Paula M. Araújo, Fernanda S. Coelho, Rajesh K. Gazara, Fabrício Almeida-Silva, Thiago M. Venancio, Clicia Grativol

**Affiliations:** 1 Centro de Biociências e Biotecnologia, Laboratório de Química e Função de Proteínas e Peptídeos, Universidade Estadual do Norte Fluminense Darcy Ribeiro, Campos dos Goytacazes, Rio de Janeiro 28013-602, Brazil; sarasm.sangi@gmail.com (S.S.); paulaaraujo.bio@gmail.com (P.M.A.); fernanda_sc00@hotmail.com (F.S.C.); raj.gzra@gmail.com (R.K.G.); fabricio_almeidasilva@hotmail.com (F.A.-S.); tmvenancio@uenf.br (T.M.V.); 2 Department of Biotechnology, Indian Institute of Technology Roorkee, Roorkee 247667, India; 3 Department of Electrical Engineering, Indian Institute of Technology Rooekee, Roorkee 247667, India

**Keywords:** *COBRA* genes, cell wall dynamics, GPI-anchored proteins, cellulose, gene duplication, transcription factors

## Abstract

The *COBRA-like* (*COBL*) gene family has been associated with the regulation of cell wall expansion and cellulose deposition. *COBL* mutants result in reduced levels and disorganized deposition of cellulose causing defects in the cell wall and inhibiting plant development. In this study, we report the identification of 24 *COBL* genes (*GmCOBL*) in the soybean genome. Phylogenetic analysis revealed that the *COBL* proteins are divided into two groups, which differ by about 170 amino acids in the N-terminal region. The *GmCOBL* genes were heterogeneously distributed in 14 of the 20 soybean chromosomes. This study showed that segmental duplication has contributed significantly to the expansion of the *COBL* family in soybean during all *Glycine*-specific whole-genome duplication events. The expression profile revealed that the expression of the paralogous genes is highly variable between organs and tissues of the plant. Only 20% of the paralogous gene pairs showed similar expression patterns. The high expression levels of some *GmCOBLs* suggest they are likely essential for regulating cell expansion during the whole soybean life cycle. Our comprehensive overview of the *COBL* gene family in soybean provides useful information for further understanding the evolution and diversification of *COBL* genes in soybean.

## 1. Introduction

The plant cell wall is a dynamic network that provides mechanical support, determines the cell shape, and controls the cell expansion [1]. The primary cell wall surrounds the entire cell, providing physical strength and extensibility to allow cell expansion, and plant growth [2]. When cell elongation ceases, the secondary cell wall is formed, and provides resistance to the tissues of the plant [3]. The cell shape and direction of cell expansion is, in part, controlled by the orientation of cellulose microfibrils [4]. Cellulose microfibrils consist of β (1–4)-linked glucan residue chains and form the major component of the cell wall [5]. Cellulose provides higher rigidity and extensibility to the cell wall. The cellulose microfibrils are synthesized by highly unstable integral plasma membrane protein complexes called rosettes. Members of the *cellulose synthase A* (*CesA*) gene family encode glycosyltransferases that play a key role in rosette synthesis [6,7].

The *COBRA-like* (*COBL*) genes belong to an essential gene family that participates in the regulation of cell wall expansion and is co-expressed with some members of the *CesA* gene family. [8,9]. The *COBLs* genes encode glycosylphosphatidylinositol (GPI)-anchored proteins with a hydrophilic region, a Cys-Cys-Val-Ser domain (CCVS), a potential N-glycosylation site, an N-terminal peptide signal, and a predicted cellulose-binding site [10]. The GPI-anchor modification sites (ω-sites) are cleaved, and the GPI anchor structure are added through an amide bond in the C-terminal region of the protein [11]. The *COBL* proteins are then secreted into Golgi vesicles and later located on the plasma membrane to modulate the cellulose chains’ assembly and crystallization [12].

*COBL* genes play an important role in cellulose biosynthesis, affect the orientation of cell expansion, reduce levels of cellulose microfibrils, and are involved in defense response [9,13,14]. The *COBL* genes were identified in several dicotyledons and monocotyledons like tomato (*Solanum lycopersicum*) [15], maize (*Zea mays*) [16,17], rice (*Oryza sativa*) [12], cotton (*Gossypium* spp.) [18], *Populus* [19], and Arabidopsis (*Arabidopsis thaliana*) [10]. Arabidopsis, maize, and rice contain 12, 11, and 9 *COBL* genes, respectively, and can be divided into two main subgroups. One of these is characterized by additional amino acids in the N-terminal region [10]. 

In Arabidopsis, *COBL* deficient plants show defects in cell expansion, lack of crystallization, and cell deposition [9]; besides they are critical for the directional growth of pollen tubes [20]. In cotton, some *COBL* genes affect the quality of cotton fibers [18]. Also, the *COBL* genes participate in the biosynthesis of the secondary cell wall. Mutants of maize *Bk2*, which encodes a *COBL* protein, have drastic changes in the composition and structure of secondary cell walls and affect stem strength [17,21]. In rice, *COBL* mutants have a reduction in cellulose content, modulate the cell wall assembly, and regulate the deposition of secondary cell wall components [12,22]. 

Soybean (*Glycine max*) is one of the most important crops in the world. The soybean genome has undergone two rounds of whole-genome duplication (WGD) about 59 and 13 million years ago (Mya) [23]. These WGDs contributed to the expansion of soybean genes and approximately 75% of the soybean genes belong to multigene families [23]. Cell wall composition is determined by the action of a large number of gene families, including *COBL* genes. Although *COBL* genes have been characterized in important crop plants, we lack the information about these genes in soybean. 

Here, we identified the full set of *COBL* sequences in the soybean genome and characterized this important plant gene family. We predict its structure, analyze ontology and duplication patterns. We investigated the expression profile of these genes in different plant organs and tissues. We found genes that appear to be essential in several stages of soybean development. 

## 2. Results

### 2.1. Identification of COBL Genes in Soybean

Using BLASTp (Basic Local Alignment Search Tool algorithms) and HMM (Hidden Markov Model) profile search, we found a total of 24 *COBL* genes in the soybean genome version 2, which were designated as *GmCOBL*1 to *GmCOBL*24, according to their chromosomal locations (Table 1). The exon-intron boundaries annotation of *COBL* genes were validated by the assembled transcripts from 1248 RNA-seq libraries [24]. All *COBL* genes listed in Table 1 showed exact match of exon-intron boundaries and the presence of a *COBL* domain in their translated peptides. The lengths of CDSs range from 630 bp to 2016 bp. Protein lengths range from 209 to 671 amino acids. Most of the *COBL* genes identified in soybean have the CCVS motif, the N-terminal signal peptide, and the local potential Ω-site. Only one gene identified, *GmCOBL*2, does not have the N-terminal signal peptide and local potential Ω-site in its structure.

### 2.2. Phylogenetic and Structural Analysis of COBL Genes in Soybean

**Figure 1:**
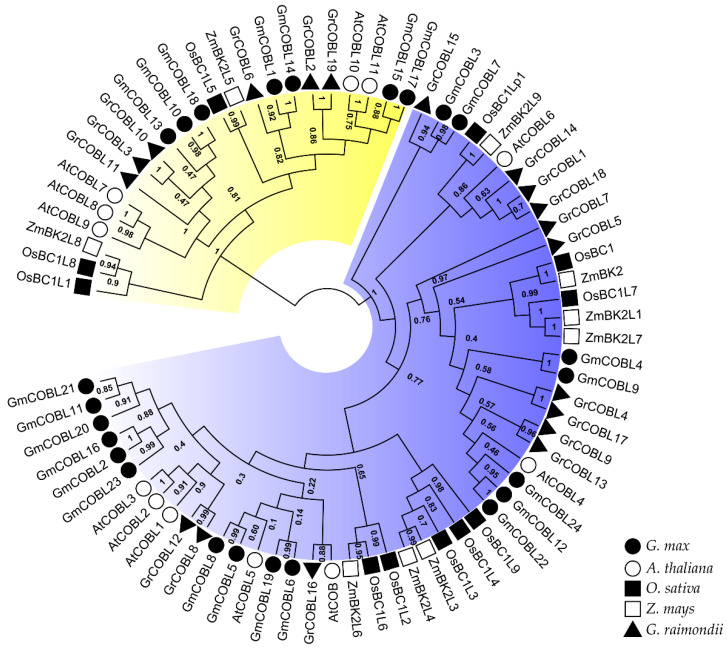
Phylogenetic relationship of *COBL* genes in soybean, Arabidopsis, rice, cotton, and maize. Multiple alignments of amino acid sequences were performed using ClustalW and the phylogenetic tree was constructed using MEGA X software by the Maximum Likelihood method. The value at the branches represents bootstrap values from 1000 replicates. Five species were represented by five different symbols. Different groups of the *COBL* family are indicated by colors. Blue is Group I and yellow Group II.

To analyze the evolutionary relationships among *COBL* proteins in soybean, we built an unrooted tree employing the Maximum Likelihood (ML) method using alignments of full-length amino acid sequences of *COBLs* from soybean, Arabidopsis, cotton, maize, and rice (Figure 1). According to the phylogenetic distribution, the *GmCOBL* proteins can be classified into two clades similar to Arabidopsis, maize, cotton, and rice [10,12,17,18]. Clade I contains 17 members of *GmCOBL* and clade II contains seven members of *GmCOBL* phylogenetically related to *AtCOB* and *AtCOBL*7, respectively. By the high bootstrap values of the internal branches, it is possible to deduce that there are real homologs with likely similar functions. Additionally, the phylogenetic tree topology reveals that protein pairs located at the terminal nodes are possible paralogs or orthologs.

**Figure 2:**
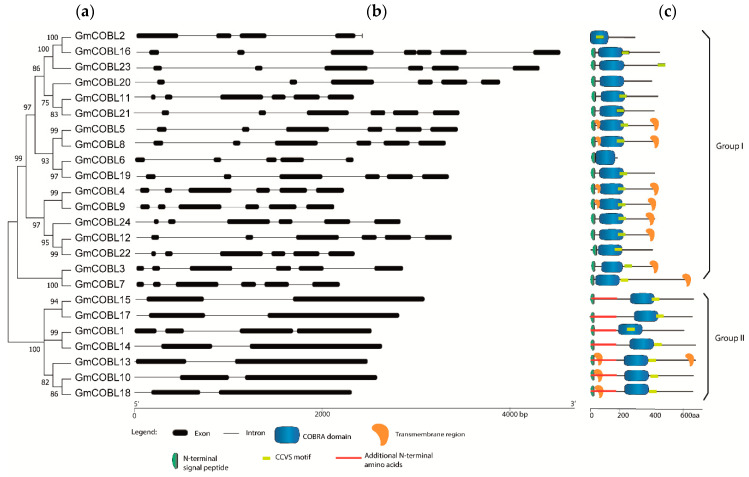
Phylogenetic relationship, gene structure, and domain architecture of *GmCOBLs* in soybean. (**a**) Phylogenetic relationship of *COBL* genes identified in soybean. (**b**) Gene structure of the 24 full-length coding *COBL* sequences in soybean. CDSs are represented with black rectangles and introns with black lines. The length of CDSs and introns can be estimated using the scale shown below. (**c**) Schematic representation of the domain architecture of the full-length *COBL* proteins. The size of domains can be estimated using the scale shown below.

To understand the structural diversity of *GmCOBL* genes and correlate the phylogenetic relationships, we analyzed the intron-exon patterns of *GmCOBLs* (Figure 2). As previously shown, *COBL* genes can be divided into two groups in the soybean genome (Figure 2a). These two groups can be differentiated by size and number of exons (Figure 2b). The average length of exons in group II is greater than the exons in group I. Most of the group I genes contain six exons each. In group II, the presence of four exons was the major feature. In general, members of the same subfamily have similar exon-intron structures. This conservation of the organization of the gene structure supports the results of the phylogenetic analysis.

To further study the diversification of the *GmCOBL* proteins, the domains, motifs, and transmembrane (TM) regions were analyzed (Figure 2c). Protein sequence analysis showed that each protein has a complete and conserved *COBRA-like* domain. Except for *GmCOBL*2, all *GmCOBL* proteins have an N-terminal signal peptide. In Group II, *GmCOBL* proteins have about 170 additional N-terminal amino acids, making the proteins in this group longer. The distribution of TM regions was variable among *GmCOBL* proteins. 

### 2.3. Distribution and Duplication of COBL Genes in the Soybean Genome 

We analyzed the distribution of the 24 *COBL* genes in the different soybean chromosomes (Figure S1). The genes were distributed in 14 of the 20 soybean chromosomes. The chromosomes 3, 10, 14, 15, 16, and 20 did not present genes. The mapping of 24 *COBL* genes in soybean chromosomes indicated a heterogeneous distribution. We also found some *COBL* genes distributed in duplicate chromosomal blocks (Figure S1).

Compared with Arabidopsis, maize, and rice, soybean presented at least twice the genes of the *COBL* family, being closer to cotton (Table 2). To better understand the expansion of *COBL* genes, we analyzed genome-wide *COBL* duplication events in the soybean genome. Most of the *COBL* genes were duplicated through WGD/segmental events except for the *GmCOBL*2 and *GmCOBL*21 genes duplicated by singleton and tandem, respectively (Table S1). This observation suggests that WGD/segmental duplication plays a vital role in the expansion of *COBL* genes in soybean. The collinear relationships of the duplicated pairs were analyzed. We identified ten paralog pairs that have a collinear relationship (Table 3). Besides, these pairs have close phylogenetic relationships (Figure 2a).

The relation of non-synonymous (Ka) and synonymous (Ks) substitution rates are important parameters used to infer the evolutionary dynamics following gene duplication. A value of Ka/Ks = 1 suggests neutral selection, a Ka/Ks value of <1 indicates negative selection and a Ka/Ks value of >1 means positive selection [25]. To calculate the evolutionary time of the *GmCOBL*, we analyzed the Ka/Ks indices for *COBL* paralogs gene pairs (Table 3). 

The Ka/Ks-ratio values for the *COBL* gene pairs ranged from 0.090 to 1.677 with an average of 0.40 (Table 3). Seven paralog pairs have low Ka/Ks ratios (<0.3) and only one >1. This indicates that most *COBL* paralogs are under strong purifying or stabilizing selection. The *GmCOBL*6-*GmCOBL*19 gene pair obtained a Ka/Ks ratio >1. This suggests that these genes are under positive natural selection, which can lead to novel biological functions. The *GmCOBL*12-*GmCOBL*22 paralog gene pair obtained the lowest Ka/Ks value (0.09) and the shortest divergence time (7.30 Mya), suggesting that this pair of genes may have maintained their functions after the duplication process.

The estimation of divergence time for 10 pairs of *COBL* paralogs showed that gene duplications occurred between 7.3 and 14.08 Mya (Table 3). This analysis suggests that the expansion of these paralogs happened during the last round of soybean WGD.

### 2.4. Tissue Expression Profiling and Biological Process

**Figure 3:**
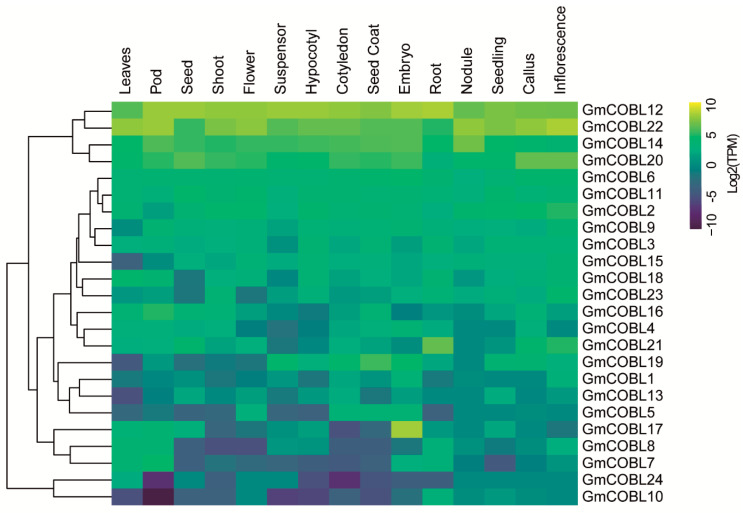
Log2 expression (TPM) of *GmCOBL* in different tissues and organs. Yellow and blue colors indicate high and low expression, respectively.

To investigate the expression patterns of *COBL* genes in soybean, transcriptome data from 15 different tissues at different developmental stages were analyzed, including Suspensor, Cotyledon, Embryo, Root, Shoot, Inflorescence, Seedling, Seed coat, Seed, Leaves, Callus, Nodule, Pod, Flower and Hypocotyl (Figure 3). 

All 24 *COBL* genes were expressed at least in one of the analyzed tissues and 15 of them were expressed in all analyzed tissues. Most *GmCOBL* genes showed distinct expression patterns. For example, *GmCOBL*10, 24, 7, and 8 showed low expression in all tissues. *GmCOBL*17 was predominantly expressed in the embryo. *GmCOBL*19 showed higher expressions only in tissues related to seeds (seed, seed coat, seedling, embryo, cotyledon, and suspensor), suggesting a key role in seed germination. *GmCOBL*12, 22, 14, and 20 showed higher expression levels in all analyzed tissues, suggesting a constitutive role of these genes throughout the soybean life. 

Some pairs of paralogous genes presented different levels of expression, while others showed a similar pattern. For example, *GmCOBL*14 showed high expression in most of the tissues analyzed, while *GmCOBL*1 showed low expression. The same occurred in *GmCOBL*3-*GmCOBL*7 and *GmCOBL*10-*GmCOBL*18 paralog pairs. These data indicate that these genes have acquired different functions after the duplication event. In contrast, the *GmCOBL*22-*GmCOBL*12 and *GmCOBL*5-*GmCOBL*8 paralog pairs showed similar expression patterns, strengthening the idea that these genes maintained the same functions after the duplication event.

To obtain more information about *GmCOBL* genes roles in different conditions and stresses, we analyzed the expression profile of all COBL genes of soybean in response to water stress, salinity, dehydration, ozone treatment, and different phytohormones using publicly available RNA-seq data (Figure S2). The expression of some *GmCOBL* genes was induced under all the different conditions and stresses. Phytohormone treatment induced the expression of 9 genes (*GmCOBL*12, 21, 18, 3, 6, 23, 11, 10 and 4) (Figure S2a). Treatment with ethylene in soybean leaves induced *GmCOBL*12, 22, 23, and 2 gene expression. 

Treatment with water-limiting and salt stress conditions on soybean roots induced *GmCOBL*12 and *GmCOBL*21 expression (Figure S2c,e). The treatment with water limitation in soybean leaves induced *GmCOBL*12 and *GmCOBL*22 expression (Figure S2d). Furthermore, flowers and soybean pods subjected to high ozone concentrations induced the expression of *GmCOBL*12 and *GmCOBL*22 (Figure S2f). This analysis shows that *GmCOBL*12 is induced in different treatments and stresses, suggesting that it is essential for cell wall maintenance.

The gene ontology (GO) enrichment analysis of 24 *GmCOBL* using AgriGO showed the biological processes in which these genes are related. The *GmCOBLs* are related to biogenesis, assembly, organization, and biological regulation of the cell wall. Also, the analyses showed a high enrichment of GO terms related to the regulation of cell growth (Figure S3). These analyses indicate that the *COBL* genes in soybean are associated with positive and negative regulatory events related to biogenesis, cell wall assembly, and cell growth.

### 2.5. Promoter Cis-Regulatory Element Analysis

Promoter cis-regulatory elements play fundamental roles in gene expression initiation and can indicate the different functions that these genes perform. To analyze the cis-regulatory elements in the promoters of the soybean *COBL* genes, we used the PlantCare database to identify the associated cis-regulatory elements.

As shown in the analysis of gene expression, the *GmCOBL* genes were induced upon different stresses and phytohormone treatment; the promoters have different cis-regulatory elements related to the response of plant hormones and response to biotic and abiotic stresses (Table S2). Of the 24 *GmCOBLs* identified, 19 (79%) had the cis-regulatory elements MYB (stress response, drought tolerance), 18 (75%) contained the element MYC (drought tolerance) and 9 (37%) had the element MBS (drought-inducibility). Also, 45% of *GmCOBL* genes promoters had the element STRE (responsiveness to thermal stress). Considering cis-regulatory elements related to plant hormone responses, ERE (ethylene-response element), TCA(methyl-jasmonate-responsive element), TGA(methyl-jasmonate-responsive element), ABRE (abscisic acid-responsive element), TATC (gibberellin-responsive element), and CGTCA (methyl-jasmonate-responsive element) were present in the promoting region in several of the *GmCOBLs*.

The *GmCOBLs* 12 and 22 showed the largest number of different cis-regulatory elements that may be related to their high expression in soybean tissues. Paralogous genes showed moderate correspondence regarding the distribution of cis-regulatory elements. For example, the paralog pair *GmCOBL*12-*GmCOBL*22 shares 20 different cis-regulatory elements while the pair *GmCOBL* 1-*GmCOBL*14 shares only 9. These data may indicate that after gene duplication, the promoters of some paralogs diverged which may have contributed to their divergent transcriptional profiles. Besides, the presence of different regulatory elements in the *GmCOBLs* promoting region indicates the participation of these genes in the development and response to hormonal variations and stress in soybean plants.

## 3. Discussion

### 3.1. Soybean Contains 24 COBL Genes

*COBL* genes are necessary for cellular expansion [9,10], grain yield [26] and are required for cellulose synthesis [8]. The *COBL* family has been studied in some plants [10,16,19,20]. However, there are no studies on the *COBL* genes in soybean. In our study, we identified a total of 24 *COBL* genes throughout the soybean genome. As described in Arabidopsis [10], maize [16], rice [12], and cotton [18], the phylogenetic analysis grouped the *GmCOBL* into two groups. Group I of the *COBL* genes in soybean contains 17 members and resembles the genes of the COB family of Arabidopsis. Group II comprises seven members and resembles *AtCOBL*7 from Arabidopsis. Group I differs from group II due to additional 170 N-terminal amino acids [10]. The difference in gene structure as well as in the number of exons and introns gives support to the division into two clades (Figure 2b). Besides, phylogenetic analysis showed that the two groups contain *COBL*s of both monocots and dicots. This analysis suggests that *COBL* family members are descendants of an ancient duplication that occurred before the separation of monocots and dicots. As a result of subsequent duplications, the number of *COBL* genes is higher in dicots.

*COBL* proteins have a predicted anchorage site for glycosylphosphatidylinositol (GPI) that is connected via an amino acid designated ω to GPI anchors [9,27]. The *COBL* proteins are then secreted to Golgi vesicles and later to the outer surface of the cell wall [10]. Most *GmCOBLs* have a signal peptide in the N-terminal region (Figure 2c). This signal peptide is necessary to direct *COBL* proteins to the Golgi apparatus. Also, most *GmCOBLs* have a potential ω-site (Table 1). These results indicate that most of *GmCOBLs* are secreted in the Golgi vesicles and can later be directed to the cell wall surface to influence cellulose deposition and cell expansion.

### 3.2. WGD Collaborated to COBL Gene Expansion in Soybean

We found more than twice as many *COBL* genes in soybean compared to Arabidopsis (Table 2). This expansion of *COBL* genes in the soybean genome may be due to segmental duplications (Table S1). Among 24 identified *COBL* genes, 22 are within segmental duplications, whereas only two are singleton or tandem. This result is consistent with previous reports where it is found that the predominant duplication in the soybean genome is segmental [28,29]. In other dicots like cotton, the predominant duplication between *COBL* genes is also segmental [18].

The soybean genome has undergone two rounds of whole-genome duplication (WGD), 13 and 59 Mya [23]. Our analysis identified three paralog pairs that derive from the first WGD and seven paralog pairs that derive from the second WGD. These data suggest that the most recent WGD duplication may be the main mechanism for the expansion and functional diversification of *COBL* genes in soybean. 

### 3.3. Expression Profiles of COBL Gene Family in Soybean Showed Functional Diversity

The expression pattern of the *GmCOBL* genes in different organs and tissues is quite heterogeneous, indicating that the different *COBL* family members in soybean differ in function. Inside the subgroup without the 170 amino acid N-terminal stretch, the *GmCOBL*12-*GmCOBL*22 paralog pair showed high and similar expression in different soybean tissues and organs (Figure 3 and Figure S2). Among the paralogous genes, *GmCOBL*12-*GmCOBL*22 showed the lowest value of Ka/Ks. Because they have shorter divergence times, these genes retained the same functions. Due to their close phylogenetic relationship, these genes are possibly orthologs of the *AtCOBL*4 gene. The *AtCOBL*4 gene modulates the assembly of cellulose microfibrils [10]. Moreover, *AtCOBL*4 is highly co-expressed with the *CESA* cellulose biosynthesis genes 4, 7, and 8 [8,30]. In rice and sorghum, the BC1 gene, ortholog of *AtCOBL*4, participates in controlling the mechanical resistance of the plant and regulates the cellulose content in the secondary cell wall [22,31]. Another ortholog of the *AtCOBL*4 gene, the *Zm*BK2 in maize, is also expressed in different tissues and participates in the deposition of cellulose on the secondary walls [17,21]. Furthermore, the *GmCOBL*12-*GmCOBL*22 gene pair had its expression regulated in response to environmental stresses and hormone treatments. Some *COBL* genes identified in *Populus* and maize have been up-regulated in response to hormonal treatments [16,19]. In Arabidopsis, only the *AtCOBL*10 and 11 genes had their expression regulated in response to stresses and hormones [16]. Together, these data suggest that the *GmCOBL*12 and 22 genes may be highly involved in response to stresses and hormones and actively participate in the cellulose deposition process in the plant cell wall, being crucial for plant development.

The paralog pair *GmCOBL*19-*GmCOBL*6 obtained the highest value of Ka/Ks and presented quite different expression patterns, strongly supporting their functional divergence after duplication. *GmCOBL*19 exhibited high expression in tissues related to seed formation and is likely the one retaining functions similar to those of *AtCOBL*2, which is necessary for the cellulose deposition in Arabidopsis seed coats [32]. Furthermore, *AtCOBL*2 plays a crucial role in controlling cellulose production in primary radial walls [33,34]. This suggests that the *GmCOBL*19 gene possibly participates in regulating the cellulose deposition process in the primary cell wall during the soybean seed formation process. 

In the second subgroup, where the members have an N-terminal stretch of 170 amino acids, the *GmCOBL*14 gene showed high expression in different tissues, except in callus and inflorescence. This gene may be involved in the cell expansion and cellulose deposition process in most soybean tissues. Besides this, this gene has a close phylogenetic relationship with the *OsBC1L*5 gene. Mutants of the *OsBC1L*5 gene prevent the germination of pollen and block male gametophyte transmission [35].

The *GmCOBL1*7 gene showed a high expression only in embryo soybean tissue. This gene can participate in cell expansion, rearrangement, and assembly of cellulose microfibrils during the germination of the soybean embryo. It has already been seen that cellulose plays a fundamental role in cell expansion during the germination of the embryonic soybean axis [36]. Moreover, some *COBL* genes have been described as differentially expressed during the germination of the soybean embryo [37]. Additionally, the *GmCOBL*17 gene showed a close phylogenetic relationship with the *AtCOBL*10. In Arabidopsis, mutations in the *COBL*10 gene have been reported to cause gametophyte sterility due to reduced pollen tube growth [20]. 

In summary, a total of 24 *COBL* genes were found in the soybean genome, being randomly distributed in its chromosomes and most of them were expanded through segmental duplications in the last WGD. The RNA-seq data provides information on the main functions of these genes. Most *GmCOBL* present functions of formation, regulation, and growth of the cellular wall in several vegetal organs whereas some members present function in specific organs. Our results indicate that the *COBL* gene family in soybean is strongly involved in cellulose biosynthesis and cell expansion regulation in different soybean tissues. In the future, this information may lead to molecular breeding related to the cell wall in this species.

## 4. Materials and Methods

### 4.1. Identification of COBL Gene Family Members in the Soybean Genome

In order to identify *COBL* genes, the *Glycine max* Wm82.a2.v1 genome database available in Phytozome v12 (phytozome.jgi.doe.gov) was searched through Basic Local Alignment Search Tool algorithms (BLASTp) [38], using Arabidopsis *COBL* protein sequences as queries [10]. We used the Hidden Markov Model (HMM) profile corresponding to the COBRA domain (PF04833) available on the Pfam database (pfam.xfam.org/) to examine the protein sequences obtained. Sequences without the COBRA domain (PF04833) were removed. The exon-intron boundaries of *COBL* genes were validated through assembled transcripts of 248 RNA-seq libraries [24]. We used GffCompare (v0.10.5) (https://ccb.jhu.edu/software/stringtie/gffcompare.shtml) to compare assembled and reference COBL transcripts. Signal peptides were verified with SignalP 5.0 [39] and GPI modifications with big-PI (http://mendel.imp.ac.at/gpi/gpi_server.html).

### 4.2. Phylogenetic Analysis

To investigate the phylogenetic relationships and molecular evolution of the COBL gene family, a multiple sequence alignment of 75 *COBL* proteins from *G. max, A. thaliana*, *G. raimondii*, *O. Sativa,* and *Z. mays* downloaded from Phytozome v12 was built using ClustalW [40]. The phylogenetic trees were built using MEGAX software [41] following the maximum likelihood method and Jones-Taylor-Thornton (JTT) model + Gamma distributed (G) with 1000 replicates. The final phylogenetic tree was visualized and edited in FigTree v1.4.3 (http://tree.bio.ed.ac.uk/software/figtree/). 

### 4.3. Gene Structure and Protein Conserved Domains and Motifs

The gene structure was built based on coding sequence, exon length and number, and intron phase in Gene Structure Display Server (GSDS) 2.0 [42]. The conserved motifs and domains prediction was performed using SMART 7 (http://smart.embl-heidelberg.de/) [43] and MEME suite [44]. The annotation of transmembrane domains was performed using TMHMM v.2.0 (cbs.dtu.dk/services/TMHMM-2.0/). 

### 4.4. Chromosome Location and Gene Duplication 

The chromosomal location of the *COBL* genes in the soybean genome was determined using PhenoGram Plot (visualization.ritchielab.psu.edu/phenograms/plot) [45]. The *COBL* genes were mapped in soybean chromosomes according to the distribution, size, beginning, and ending information of the soybean genome database deposited in Phytozome 12. Syntenic information of soybean was downloaded from the Plant Genome Duplication Database. The Multiple Collinearity Scan toolkit (MCScanX) [46] with default parameter was employed to identify duplication events and analyze the collinearity relationships. 

### 4.5. Estimation of Non-Synonymous and Synonymous Substitution Rates and Evaluation Divergence Time

MCScanX was used to investigate the non-synonymous (Ka) and synonymous substitution (Ks) rate of syntenic gene pairs. The duplication time (million years ago, Mya) of each gene pair was estimated using Ks rate of λ substitutions per synonymous site per year, as the formula T = Ks/2λ (λ = 6.5 × 10^−9^) [47]. 

### 4.6. Expression Profile Analysis of Soybean COBL Genes

To analyze the expression pattern of *COBL* genes in soybean, we used RNA-seq data from 1248 libraries available at Soybean Expression Atlas [24]. Gene expression was estimated in Transcripts Per Million (TPM). TPM values were transformed to log2 and displayed in the form of heatmaps. Heatmaps of normalized expression were generated in R using the heatmap.2 function available in the gplots package. The AgriGO tool (http://bioinfo.cau.edu.cn/agriGO/index.php) [48] was used for gene ontology analysis. 

### 4.7. Putative Promoter Sequence Analysis

The 1000 bp upstream sequence from the start codon for each *GmCOBL* identified was retrieved from genomic DNA sequences. The upstream sequences were analyzed for the identification of cis-regulatory elements important for gene expression using PlantCare (bioinformatics.psb.ugent.be/webtools/plantcare/html) [49].

## Figures and Tables

**Table 1:** Summary of *COBRA-like* (*COBLs*) gene family members identified in soybean.

Gene Name	Gene Locus	CDS Length (bp ^1^)	Protein Length (aa ^2^)	Exon Number	Transcript Number	CCVS Motif	N-terminal Signal Peptide	Potential ω- Site Position
*GmCOBL*1	Glyma.01G240200	1800	599	4	1	Yes	yes	N(573)
*GmCOBL*2	Glyma.02G196100	1110	369	5	1	Yes	no	none
*GmCOBL*3	Glyma.04G006100	1344	447	6	1	Yes	yes	N(418)
*GmCOBL*4	Glyma.04G160000	1377	458	6	1	Yes	yes	N(429)
*GmCOBL*5	Glyma.04G160100	1371	456	6	1	Yes	yes	P(425)
*GmCOBL*6	Glyma.05G019100	630	209	5	1	No	yes	none
*GmCOBL*7	Glyma.06G005800	1338	445	6	1	Yes	yes	G(420)
*GmCOBL*8	Glyma.06G205400	1371	456	6	2	Yes	yes	P(425)
*GmCOBL*9	Glyma.06G205500	1377	458	6	1	Yes	yes	N(429)
*GmCOBL*10	Glyma.07G243200	1932	643	2	1	Yes	yes	V(617)
*GmCOBL*11	Glyma.08G249700	1347	448	6	1	Yes	yes	N(422)
*GmCOBL*12	Glyma.08G249800	1296	431	6	2	Yes	yes	N(408)
*GmCOBL*13	Glyma.09G039900	1953	650	2	1	Yes	yes	S(625)
*GmCOBL*14	Glyma.11G003300	1956	651	2	1	Yes	yes	N(628)
*GmCOBL*15	Glyma.12G213400	2016	671	2	1	Yes	yes	S(647)
*GmCOBL*16	Glyma.13G053300	1377	458	6	2	Yes	yes	N(432)
*GmCOBL*17	Glyma.13G288300	2001	666	2	1	Yes	yes	S(642)
*GmCOBL*18	Glyma.17G030700	1935	644	2	1	Yes	yes	S(615)
*GmCOBL*19	Glyma.17G080600	1365	454	6	1	Yes	yes	A(423)
*GmCOBL*20	Glyma.18G271900	1263	420	6	3	No	yes	none
*GmCOBL*21	Glyma.18G272000	1347	448	6	1	Yes	yes	A(423)
*GmCOBL*22	Glyma.18G272100	1296	431	6	2	Yes	yes	N(408)
*GmCOBL*23	Glyma.19G033500	1365	454	6	1	Yes	yes	N(428)
*GmCOBL*24	Glyma.19G033600	1296	431	6	2	Yes	yes	N(408)

^1^ base pairs; ^2^ amino acids.

**Table 2:** *COBRA-like* gene distribution among species.

Species Name	Total Number of *COBRA-Like* Genes	Haploid Genome Size (Mb ^1^)	Chromosomes (*n* ^2^)	Reference
*Glycine max*	24	1100	10	Our study
*Gossypium raimondii*	19	880	13	(Niu, 2015)
*Arabidopsis thaliana*	12	135	5	(Roudier, 2002)
*Oryza sativa*	11	500	12	(Li et al., 2003)
*Zea mays*	9	2400	10	(Brady et al., 2007)

^1^ megabases. ^2^ haploid chromosome number.

**Table 3:** The Ka/Ks-ratio values of the paralogs *GmCOBL* pairs. Mya—million years ago.

Gene 1	Gene 2	Ka	Ks	Ka/Ks	Duplication Date (Mya)
*GmCOBL*1	*GmCOBL*14	0.051	0.104	0.491	8.55
*GmCOBL*3	*GmCOBL*7	0.026	0.112	0.233	9.17
*GmCOBL*4	*GmCOBL*9	0.021	0.157	0.131	12.84
*GmCOBL*5	*GmCOBL*8	0.019	0.092	0.204	7.55
*GmCOBL*6	*GmCOBL*19	0.224	0.134	1.677	10.97
*GmCOBL*10	*GmCOBL*18	0.017	0.139	0.124	11.38
*GmCOBL*11	*GmCOBL*21	0.110	0.190	0.579	9.02
*GmCOBL*12	*GmCOBL*22	0.008	0.089	0.090	7.30
*GmCOBL*15	*GmCOBL*17	0.036	0.172	0.210	14.08
*GmCOBL*16	*GmCOBL*23	0.031	0.140	0.224	11.49

## Data Availability

The data presented in this study are available in article and supplementary materials.

## Supplementary Materials

The following data is available online at https://www.mdpi.com/2223-7747/10/1/167/s1, Figure S1: Distribution of the *GmCOBL* in soybean chromosomes. Only the chromosomes that contain *GmCOBL* genes are represented. The chromosome number is shown above each chromosome. Genes names marked in the same color are a pair of paralogs. Circled boxes indicate the gene blocks. Table S1: List of duplication events of the *GmCOBL* gene family in soybean. Figure S2. Log2 expression (TPM) of *GmCOBL* in adverse conditions. Yellow and blue colors indicate high and low expression respectively. (**a**) Two-week-old soybean roots grown in a dark setting and not treated with any phytohormone, treated with auxin, treated with jasmonic acid and ethylene. (**b**) soybean leaves explants exposed to ethylene for 0 to 72 h. (**c**) soybean roots under water-limiting conditions. (**d**) soybean leaves after 0, 6, 12, and 24 h under water deficit. (**e**) soybean roots after application of 100 mM NaCl solution. (**f**) soybean pod and flower exposed to elevated ozone concentrations. Figure S3: Functional and biological process category distribution of *GmCOBL* genes in soybean. The boxes on the graph represent GO terms labeled by their ID. term definition and statistical information. The degree of color saturation in a box is positively correlated with the level of enrichment of the term. Solid lines mean a connection. solid green lines mean negative regulation and dashed lines mean a significant node. Table S2: Cis-regulatory elements identified in the promoter of the *GmCOBLs*.

## References

[1] Cosgrove D.J. (2016). Plant cell wall extensibility: Connecting plant cell growth with cell wall structure, mechanics, and the action of wall-modifying enzymes. J. Exp. Bot..

[2] Carpita N.C., Gibeaut D.M. (1993). Structural models of primary cell walls in flowering land plants—Consistency of molecular structure with the physical properties of the walls during growth. Plant J..

[3] Kumar M., Campbell L., Turner S. (2016). Secondary cell walls: Biosynthesis and manipulation. J. Exp. Bot..

[4] Cosgrove D.J. (2015). Plant expansins: Diversity and interactions with plant cell walls. Curr. Opin. Plant Biol..

[5] Brown R.M. (2004). Cellulose Structure and Biosynthesis: What is in Store for the 21st Century?. J. Polym. Sci. Part A Polym. Chem..

[6] Malcolm Brown R., Saxena I.M., Kudlicka K. (1996). Cellulose biosynthesis in higher plants. Trends Plant Sci..

[7] Suzuki S., Li L., Sun Y.-H., Chiang V.L. (2006). The Cellulose Synthase Gene Superfamily and Biochemical Functions of Xylem-Specific Cellulose Synthase-Like Genes in Populus trichocarpa. Plant Physiol..

[8] Persson S., Wei H., Milne J., Page G.P., Somerville C.R. (2005). Identification of genes required for cellulose synthesis by regression analysis of public microarray data sets. Proc. Natl. Acad. Sci. USA.

[9] Schindelman G., Morikami A., Jung J., Baskin T.I., Carpita N.C., Derbyshire P., McCann M.C., Benfey P.N. (2001). COBRA encodes a putative GPI-anchored protein, which is polarly localized and necessary for oriented cell expansion in arabidopsis. Genes Dev..

[10] Roudier F. (2002). The COBRA Family of Putative GPI-Anchored Proteins in Arabidopsis. A New Fellowship in Expansion. Plant Physiol..

[11] Borner G.H.H., Sherrier D.J., Stevens T.J., Arkin I.T., Dupree P. (2002). Prediction of Glycosylphosphatidylinositol-Anchored Proteins in Arabidopsis. A Genomic Analysis. Plant Physiol..

[12] Liu L., Shang-Guan K., Zhang B., Liu X., Yan M., Zhang L., Shi Y., Zhang M., Qian Q., Li J. (2013). Brittle Culm1, a COBRA-Like Protein, Functions in Cellulose Assembly through Binding Cellulose Microfibrils. PLoS Genet..

[13] Bahari M.N.A., Sakeh N.M., Abdullah S.N.A., Ramli R.R., Kadkhodaei S. (2018). Transciptome profiling at early infection of Elaeis guineensis by Ganoderma boninense provides novel insights on fungal transition from biotrophic to necrotrophic phase. BMC Plant Biol..

[14] Niu E., Fang S., Shang X., Guo W. (2018). Ectopic expression of GhCOBL9A, a cotton glycosyl-phosphatidyl inositol-anchored protein encoding gene, promotes cell elongation, thickening and increased plant biomass in transgenic Arabidopsis. Mol. Genet. Genom..

[15] Cao Y., Tang X., Giovannoni J., Xiao F., Liu Y. (2012). Functional characterization of a tomato COBRA-like gene functioning in fruit development and ripening. BMC Plant Biol..

[16] Brady S.M., Song S., Dhugga K.S., Rafalski J.A., Benfey P.N. (2007). Combining Expression and Comparative Evolutionary Analysis. The COBRA Gene Family. Plant Physiol..

[17] Ching A., Dhugga K.S., Appenzeller L., Meeley R., Bourett T.M., Howard R.J. (2006). Brittle stalk 2 encodes a putative glycosylphosphatidylinositol- anchored protein that affects mechanical strength of maize tissues by altering the composition and structure of secondary cell walls. Planta.

[18] Niu E., Shang X., Cheng C., Bao J., Zeng Y., Cai C., Du X., Guo W. (2015). Comprehensive analysis of the COBRA-like (COBL) gene family in Gossypium identifies two COBLs potentially associated with fiber quality. PLoS ONE.

[19] Ye X., Kang B.G., Osburn L.D., Cheng Z.M. (2009). The COBRA gene family in Populus and gene expression in vegetative organs and in response to hormones and environmental stresses. Plant Growth Regul..

[20] Li S., Ge F., Xu M., Zhao X., Huang G., Zhou L., Wang J., Kombrink A. (2013). Arabidopsis COBRA-LIKE 10, a GPI-anchored protein, mediates directional growth of pollen tubes. Plant J..

[21] Sindhu A., Langewisch T., Olek A., Multani D.S., McCann M.C., Vermerris W., Carpita N.C., Johal G. (2007). Maize Brittle stalk2 encodes a COBRA-like protein expressed in early organ development but required for tissue flexibility at maturity. Plant Physiol..

[22] Sato K., Suzuki R., Nishikubo N., Takenouchi S., Ito S., Nakano Y., Nakaba S., Sano Y., Funada R., Kajita S. (2010). Isolation of a novel cell wall architecture mutant of rice with defective Arabidopsis COBL4 ortholog BC1 required for regulated deposition of secondary cell wall components. Plant Signal. Behav..

[23] Schmutz J., Cannon S.B., Schlueter J., Ma J., Mitros T., Nelson W., Hyten D.L., Song Q., Thelen J.J., Cheng J. (2010). Genome sequence of the palaeopolyploid soybean. Nature.

[24] Machado F.B., Moharana K.C., Almeida-Silva F., Gazara R.K., Pedrosa-Silva F., Coelho F.S., Grativol C., Venancio T.M. (2020). Systematic analysis of 1298 RNA-Seq samples and construction of a comprehensive soybean (Glycine max) expression atlas. Plant J..

[25] Hurst L.D. (2002). The Ka/Ks ratio: Diagnosing the form of sequence evolution. Trends Genetics..

[26] Hochholdinger F., Wen T.J., Zimmermann R., Chimot-Marolle P., Da Costa E., Silva O., Bruce W., Lamkey K.R., Wienand U., Schnable P.S. (2008). The maize (Zea mays L.) roothairless3 gene encodes a putative GPI-anchored, monocot-specific, COBRA-like protein that significantly affects grain yield. Plant J..

[27] Brown D., Waneck G.L. (1992). Glycosyl-phosphatidylinositol-anchored membrane proteins. J. Am. Soc. Nephrol..

[28] Pagel J., Walling J.G., Young N.D., Shoemaker R.C., Jackson S.A. (2004). Segmental duplications within the Glycine max genome revealed by fluorescence in situ hybridization of bacterial artificial chromosomes. Genome.

[29] Shoemaker R.C., Schlueter J., Doyle J.J. (2006). Paleopolyploidy and gene duplication in soybean and other legumes. Curr. Opin. Plant Biol..

[30] Taylor N.G. (2008). Cellulose biosynthesis and deposition in higher plants. New Phytol..

[31] Li P., Liu Y., Tan W., Chen J., Zhu M., Lv Y., Liu Y., Yu S., Zhang W., Cai H. (2018). Brittle Culm 1 Encodes a COBRA-Like Protein Involved in Secondary Cell Wall Cellulose Biosynthesis in Sorghum. Plant Cell Physiol..

[32] Ben-Tov D., Abraham Y., Stav S., Thompson K., Loraine A., Elbaum R., de Souza A., Pauly M., Kieber J.J., Harpaz-Saad S. (2015). COBRA-LIKE2, a member of the glycosylphosphatidylinositol-anchored COBRA-LIKE family, plays a role in cellulose deposition in arabidopsis seed coat mucilage secretory cells. Plant Physiol..

[33] Ben-Tov D., Idan-Molakandov A., Hugger A., Ben-Shlush I., Günl M., Yang B., Usadel B., Harpaz-Saad S. (2018). The role of COBRA-LIKE 2 function, as part of the complex network of interacting pathways regulating Arabidopsis seed mucilage polysaccharide matrix organization. Plant J..

[34] Francoz E., Ranocha P., Burlat V., Dunand C. (2015). Arabidopsis seed mucilage secretory cells: Regulation and dynamics. Trends Plant Sci..

[35] Dai X., You C., Wang L., Chen G., Zhang Q., Wu C. (2009). Molecular characterization, expression pattern, and function analysis of the OsBC1L family in rice. Plant Mol. Biol..

[36] Sangi S., Santos M.L.C., Alexandrino C.R., Da Cunha M., Coelho F.S., Ribeiro G.P., Lenz D., Ballesteros H., Hemerly A.S., Venâncio T.M. (2019). Cell wall dynamics and gene expression on soybean embryonic axes during germination. Planta.

[37] Bellieny-Rabelo D., de Oliveira E.A.G., Ribeiro E. (2016). da S.; Costa, E.P.; Oliveira, A.E.A.; Venancio, T.M. Transcriptome analysis uncovers key regulatory and metabolic aspects of soybean embryonic axes during germination. Sci. Rep..

[38] Altschul S.F., Madden T.L., Schäffer A.A., Zhang J., Zhang Z., Miller W., Lipman D.J. (1997). Gapped BLAST and PSI-BLAST: A new generation of protein database search programs. Nucleic Acids Res..

[39] Almagro Armenteros J.J., Tsirigos K.D., Sønderby C.K., Petersen T.N., Winther O., Brunak S., von Heijne G., Nielsen H. (2019). SignalP 5.0 improves signal peptide predictions using deep neural networks. Nat. Biotechnol..

[40] Thompson J.D., Gibson T.J., Higgins D.G. (2003). Multiple Sequence Alignment Using ClustalW and ClustalX. Curr. Protoc. Bioinform..

[41] Kumar S., Stecher G., Li M., Knyaz C., Tamura K. (2018). MEGA X: Molecular evolutionary genetics analysis across computing platforms. Mol. Biol. Evol..

[42] Hu B., Jin J., Guo A.Y., Zhang H., Luo J., Gao G. (2015). GSDS 2.0: An upgraded gene feature visualization server. Bioinformatics.

[43] Letunic I., Doerks T., Bork P. (2011). SMART 7: Recent updates to the protein domain annotation resource. Nucleic Acids Res..

[44] Bailey T.L., Johnson J., Grant C.E., Noble W.S. (2015). The MEME Suite. Nucleic Acids Res..

[45] Wolfe D., Dudek S., Ritchie M.D., Pendergrass S.A. (2013). Visualizing genomic information across chromosomes with PhenoGram. BioData Min..

[46] Wang Y., Tang H., DeBarry J.D., Tan X., Li J., Wang X., Lee T., Jin H., Marler B., Guo H. (2012). MCScanX: A toolkit for detection and evolutionary analysis of gene synteny and collinearity. Nucleic Acids Res..

[47] Lynch M., Coneryz J.S. (2000). The Evolutionary Fate and Consequences of Duplicate Genes. Science.

[48] Du Z., Zhou X., Ling Y., Zhang Z., Su Z. (2010). agriGO: A GO analysis toolkit for the agricultural community. Nucleic Acids Res..

[49] Lescot M., Déhais P., Thijs G., Marchal K., Moreau Y., Van De Peer Y., Rouzé P., Rombauts S. (2002). PlantCARE, a database of plant cis-acting regulatory elements and a portal to tools for in silico analysis of promoter sequences. Nucleic Acids Res..

