# Supplementary figures and images for: Genome-Wide Analysis of the COBRA-Like Gene Family Supports Gene Expansion through Whole-Genome Duplication in Soybean (Glycine max)
Source: Plants (Basel). 2021 Jan 16;10(1):167. doi: 10.3390/plants10010167 (PMC7830662; doi:10.3390/plants10010167)

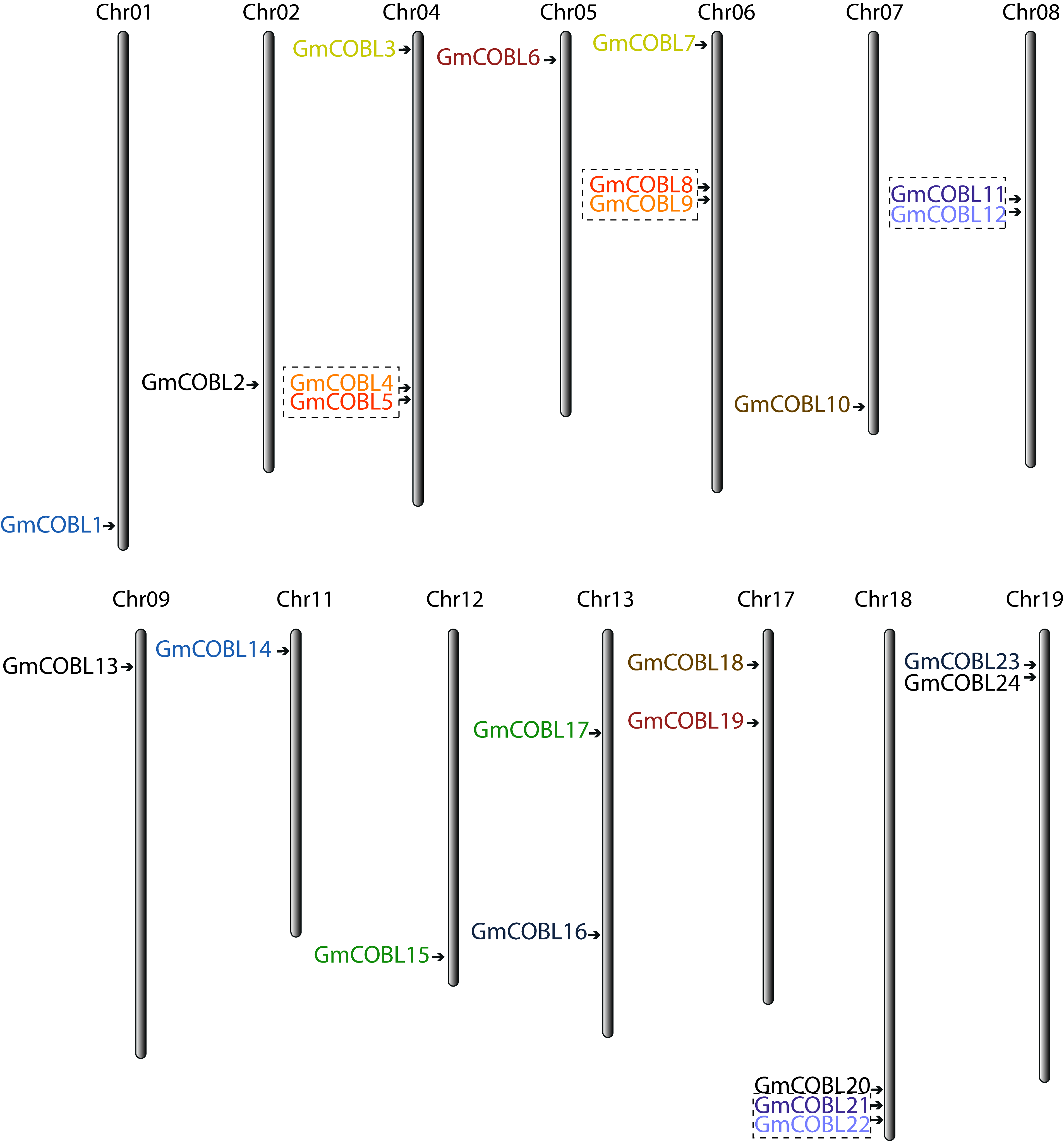

Supplement: Supplementary file 1 [file plants-10-00167-s001.zip › Supplementary files/Figure S1.tif]

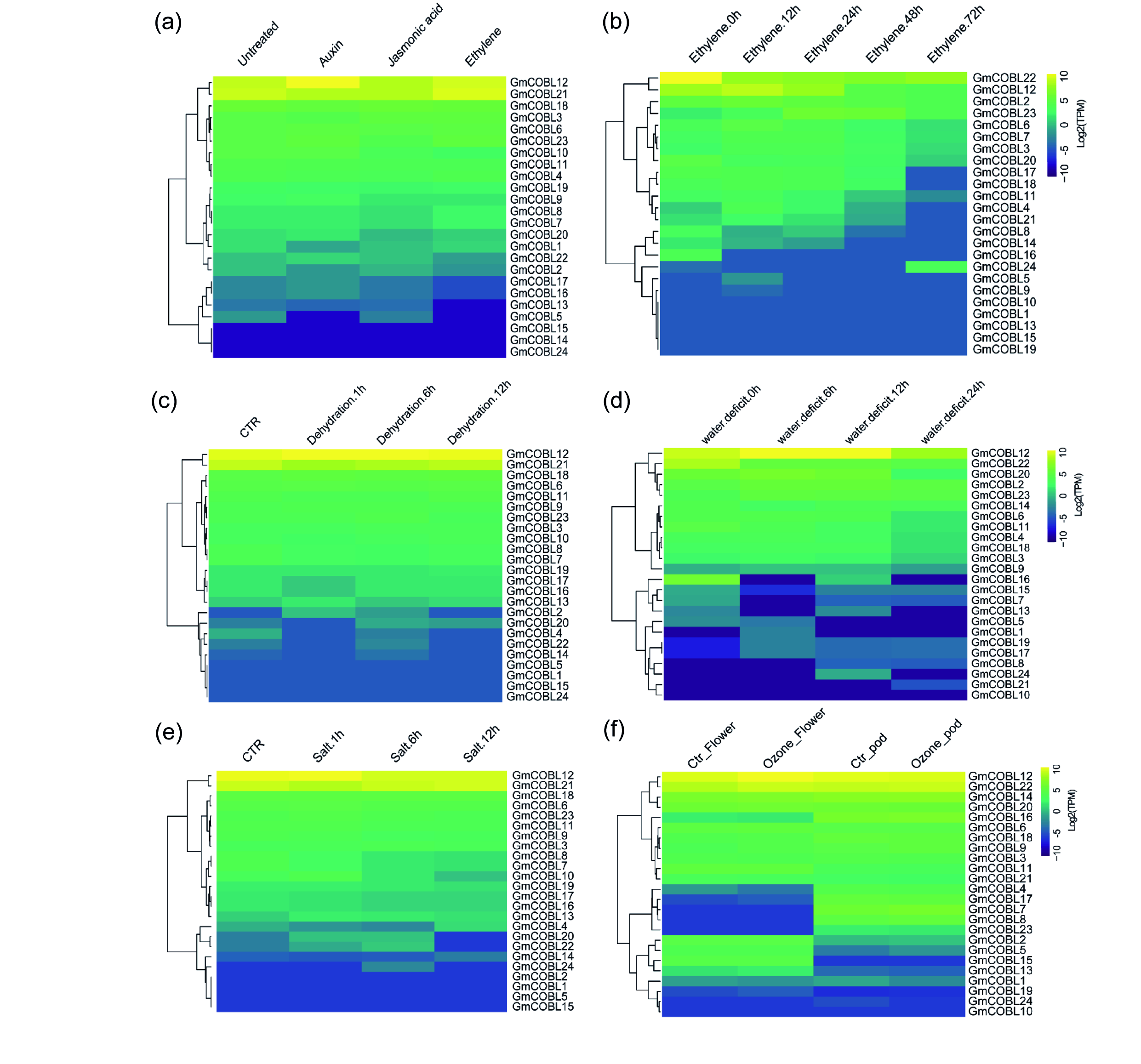

Supplement: Supplementary file 1 [file plants-10-00167-s001.zip › Supplementary files/Figure S2.tif]

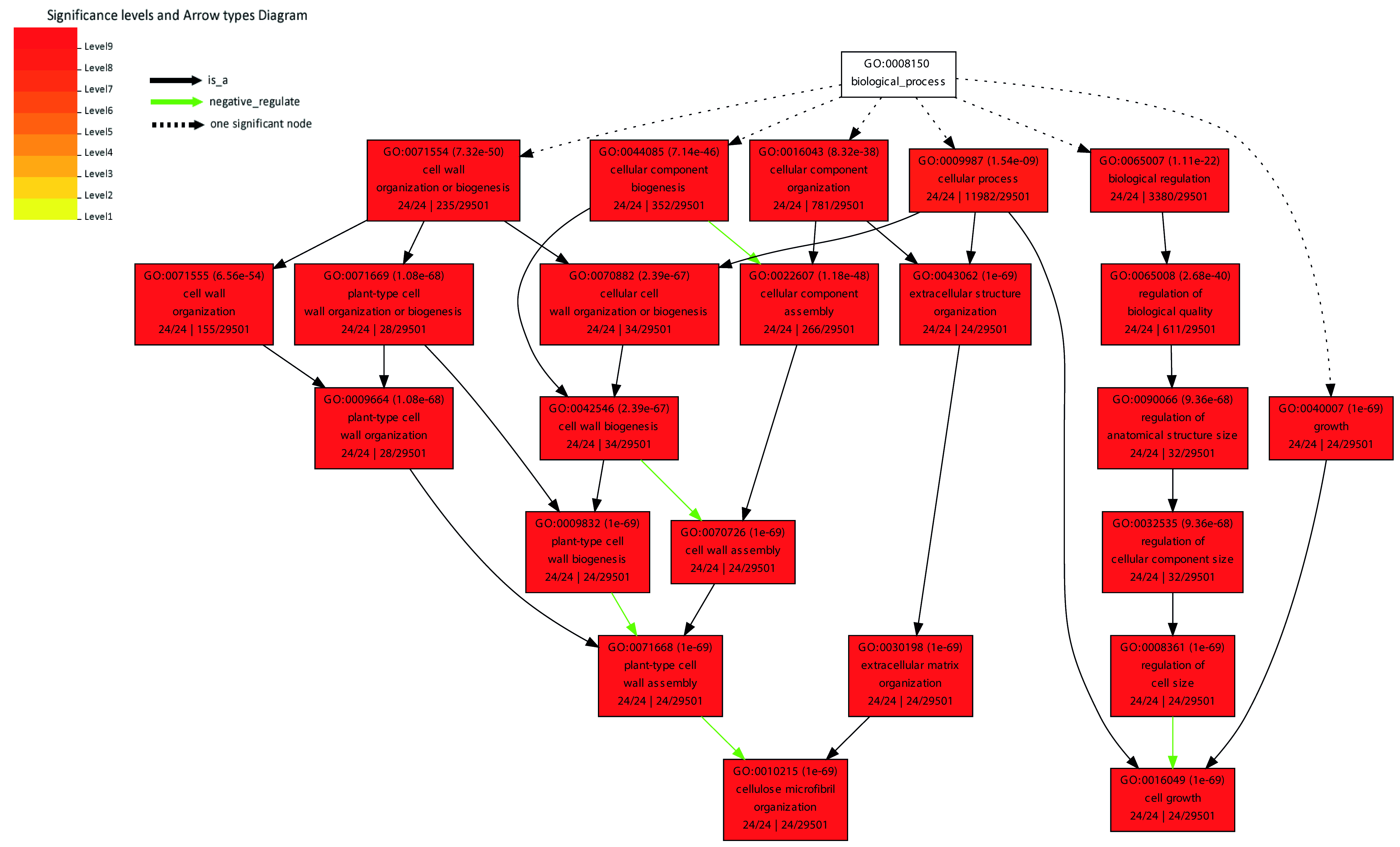

Supplement: Supplementary file 1 [file plants-10-00167-s001.zip › Supplementary files/Figure S3.tif]
